# Insight into the Molecular Mechanism of Flower Color Regulation in *Rhododendron latoucheae* Franch: A Multi-Omics Approach

**DOI:** 10.3390/plants12162897

**Published:** 2023-08-08

**Authors:** Peng Xiao, Hui Zhang, Qiulin Liao, Ninghua Zhu, Jiaao Chen, Lehan Ma, Minhuan Zhang, Shouyun Shen

**Affiliations:** 1 College of Landscape Architecture, Central South University of Forestry and Technology, Changsha 410004, China; 2 Hunan Provincial Big Data Engineering Technology Research Center of Natural Reserve and Landscape Resource, Changsha 410004, China; 3 Institute of Human Settlements and Green Infrastructure, Central South University of Forestry and Technology, Changsha 410083, China; 4 College of Forestry, Central South University of Forestry and Technology, Changsha 410004, China

**Keywords:** *Rhododendron latoucheae*, flower color regulation, metabolism group, transcriptome, flavonoids

## Abstract

*Rhododendron latoucheae* Franch. (*R. latoucheae*) is a valuable woody plant known for its high ornamental value. While purple flowers are a distinct and attractive variant phenotype of *R. latoucheae*, the underlying mechanism regulating its flower color is still poorly understood. To investigate the molecular regulatory mechanism responsible for the variation in flower color, we selected plants with white-pink and purple petals as the object and conducted analyses of metabolites, key genes, and transcription factors associated with flower color. A combined metabolome–transcriptome analysis was performed, and the expression of key genes was subsequently verified through qRT-PCR experiments. The results of our study demonstrated a significant enrichment of differential metabolites in the flavonoid metabolic pathway. Changes in anthocyanin content followed the same trend as the observed flower color variations, specifically showing significant correlations with the contents of malvidin-3-O-glucoside, dihydromyricetin, gallocatechin, and peonidin-3-O-glucoside. Furthermore, we identified three key structural genes (*F3GT1*, *LAR*, *ANR*) and four transcription factors (bHLH130, bHLH41, bHLH123, MYB4) that are potentially associated with the biosynthesis of flavonoid compounds, thereby influencing the appearance of purple flower color in *R. latoucheae*.

## 1. Introduction

Flower color is an important ornamental feature of garden plants and has a significant impact on their overall aesthetic value. Several factors, including pigment composition, pH, cell shape, and co-pigmentation, are associated with petal coloration [1]. However, floral pigments serve as the foundational material for petal coloration and represent the most influential factor in this regard [2]. The main known plant pigments include flavonoids, alkaloids, and carotenoids. Betalains are found in only a few plant species and can give petals a red color, but cannot coexist with anthocyanins. On the other hand, carotenoids can produce red, orange, and yellow petals [3]. Flavonoids are primarily distributed in the vesicles of plants as glycosides, particularly anthocyanosides, which contribute to a wide variety of petal colors [4]. Among them, betalains and carotenoids mainly give the plant a reddish color, while flavonoids are one of the most important substances that produce flower color in plants. Numerous studies have demonstrated that flavonoid metabolites play a dominant role in regulating flower color in plants. Flavonoids are secondary metabolites with diverse functions, including attracting pollinators, protecting against UV damage, and assisting in transport regulation [5]. Within the group of flavonoids, anthocyanins, chalcones, aurones, and certain flavonols are involved in flower coloration. Among these, anthocyanins are present in more than 90% of angiosperms and significantly influence flower color, including shades of purple, red, and blue [6,7]. The biosynthetic pathway of anthocyanins is highly conserved across plant species [8]. Techniques for regulating flower color based on anthocyanins have been extensively developed [9], and related studies focusing on the regulation of flower color through the flavonoid biosynthetic pathway are underway [4,10].

As an important ornamental plant, *R. latoucheae* exhibits attractive fragrance, a large number of flowers, and high ornamental value. However, its flower color tends to be light and single, mostly in shades of white and pink, which negatively impacts its overall ornamental value. Previous research has revealed that the flower color of most plants in the *Rhododendron* family is determined by flavonoids, particularly anthocyanins, while flavonols can serve as auxiliary color-presenting compounds. Notably, the petals of *Rhododendron pulchrum* primarily contain anthocyanins and flavonols, contributing to their coloration [11]. The absence of pelargonidin derivatives in the *Rhododendron* family is responsible for the lack of red azaleas [12]. White mallow azaleas contain small amounts of delphinidin and cyanidin, whereas the petals of pink mallow azaleas have six times more cyanidin-3-O-rutinoside than those of purple mallow azaleas [13]. Most studies on *Rhododendron* plants have focused on the extraction of volatile components and triterpenoids from ocimene [14], genetic diversity [15], and translocation breeding and adversity stress [16]. However, none of these studies has investigated the metabolite composition in petals or explored the content of metabolites at different developmental stages, nor have they elucidated the mechanisms of gene regulation associated with flower color. Consequently, there is a significant knowledge gap regarding the molecular regulation mechanisms of flower color in *R. latoucheae*. Conducting research on this subject will provide crucial theoretical support for breeding new varieties with enhanced ornamental value.

Therefore, our study focused on key research questions, including the differences in color characteristics among different petals of *R. latoucheae*, the composition of floral pigments, the identification of key color-presenting substances in purple petals, and the identification of key genes involved in floral color regulation. The specific objectives of our study were (i) to determine the key metabolites responsible for the differences in flower color between white, pink, and purple petals, (ii) to identify the genes that play a role in regulating purple flower color, and (iii) to elucidate the regulatory relationship between genes and metabolites in flower color formation.

Firstly, we selected plants with the most distinct flower colors by characterizing specimens with different flower colors. Secondly, we measured the content of floral pigments in petals of different flower color samples throughout the flowering period. Histological analysis was performed to identify the key metabolites that influence flower color. Additionally, we sequenced the transcriptome of different flower color samples at various developmental stages using RNA-seq to identify key genes associated with flower color formation. The association between key metabolites and flower color regulation was investigated through histological analysis. Furthermore, the expression of key genes was validated by sequencing them at different developmental stages using RNA-seq. By combining histological analysis, we aimed to uncover the causes of flower color formation and establish the molecular regulatory network of anthocyanin. Finally, the expression of key genes was verified to validate their involvement in flower color formation.

## 2. Materials and Methods

### 2.1. Materials

Through field research, we discovered that the majority of antler azaleas exhibit white-pink flowers, while a small number display purple flowers. In 2013, we initiated the introduction of *R. latoucheae* and transplanted them to an open-field nursery located in Chaling County, Zhuzhou City, Hunan Province. The nursery’s coordinates are 26°40′7″ N, 113°27′ E, with an elevation of 158 m above sea level. The soil type in the nursery is quaternary laterite.

**Figure 1:**
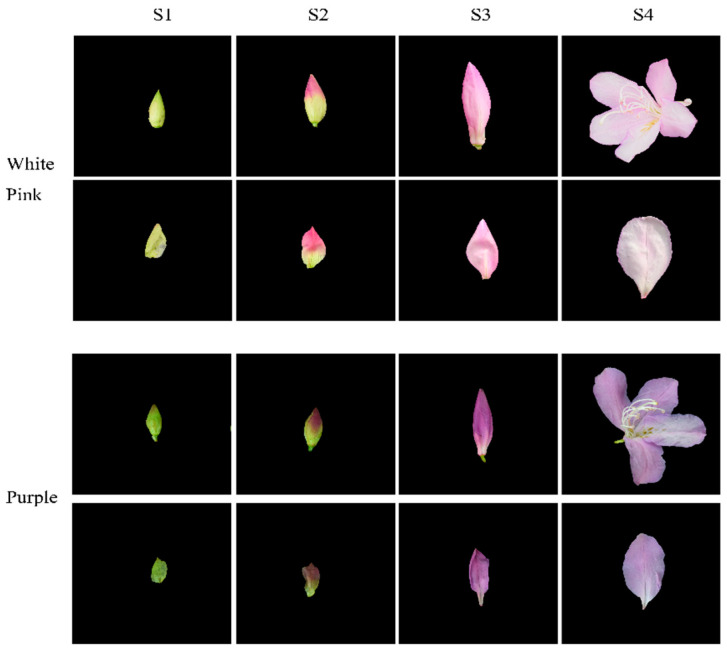
Phenotypic analysis of the development process of white-pink and purple petal of *R. latoucheae*. The development stage S1 represents the full green stage, the length of the flower bud is less than 2 cm, the flower bud is long cone type, and the petals are chartreuse. S2 represents the blooming stage, the length of the flower bud is 4–5 cm, the flower bud is cone type, and the petals are 50% colored. S3 represents the early flowering stage, with a bud length of >6 cm and fully colored petals. S4 represents the blooming period, with the petals fully blooming.

Based on phenotypic observations, we selected white-pink and purple plants within the nursery. Petals were collected at four different developmental stages during the observation period in March–April 2021. The selection of flowering periods was determined by referring to Yang’s study and conducting phenological observations for this particular study [17]. Petals from the following four stages were chosen for analysis: (1) the all-green period (S1), characterized by long and conical flower buds with uncolored and yellowish-green petals; (2) the reddish period (S2), featuring expanded and conical flower buds with 50% colored petals and exposed perianth tips; (3) the early flowering period (S3), marked by fully colored but unexpanded petals; and (4) the blooming period (S4), where petals were fully expanded (Figure 1). For each color test, eight groups with three replicates were established. The collected samples were stored in a liquid nitrogen bottle at −80 °C, with half of each sample used for metabolomic experiments and the other half for transcriptomic experiments.

### 2.2. Key Metabolite Extraction and Analytical Methods

Metabolite extraction was performed using UPLC-MS/MS (ultraperformance liquid chromatography and tandem mass spectrometry) with the following steps.

The petals were dried and ground with a grinder (MM400, Retsch, Haan, Germany) together with zirconia beads for 1.5 min. Then, 100 mg of powder was measured and dissolved in 1.0 mL of aqueous solution containing 70% methanol. The solution was stored at 4 °C overnight with vortexing performed three times. After centrifugation for 10 min, the supernatant was absorbed, filtered through a microporous membrane, stored in an injection vial, and subsequently analyzed using an LC-MS/MS system [18], Equal amounts of each sample were taken and mixed as quality control samples (QC).

The UPLC main parameters included a column UPLC HSS T3 (1.8 μm, 2.1 mm × 100 mm) with a column temperature set to 40 °C and an injection volume of 2 μL. The mobile phase A consisted of aqueous ultrapure water with 0.04% acetic acid, while the mobile phase B was acetonitrile with 0.04% acetic acid. The determination of total flavonoids followed the method of Bengtsson [19], total anthocyanins were determined using the method by King [20], and carotenoids were determined based on the method by Rassadina [21]. For sample preparation, 0.1 g of sample was taken and ground with 60% acidified ethanol. The mixture was transferred to a centrifuge tube and brought up to a volume of 20 mL. The centrifuge tube was then centrifuged at 10,000 rpm for 15 min, followed by sonication for 30 min, and finally brought up to a volume of 20 mL.

The first step involved data preprocessing, followed by qualitative and quantitative analysis of metabolites. Subsequently, sample quality control analysis was performed based on the metabolite content in each sample. The OPLS-DA model was used to screen for differential metabolites, and the KEGG database (Kyoto Encyclopedia of Genes and Genomes) was utilized for enrichment analysis and trend analysis to identify key metabolites associated with floral color. The main software used for analysis included Analyst 1.6.1 and Short Time-Series Expression Miner (v1.2.1). The main databases utilized were MassBank, KNApSAcK, MoToDB, and the METLIN database (metlin.scripps.edu, accessed on 15 May 2022).

### 2.3. Key Gene and KEY Pathway Extraction, Detection and Analysis Methods

Key genes were mainly screened from the transcriptome, and the key pathways and key genes affecting flower color were detected by using relevant analysis methods. The detection and analysis methods are as follows.

First, key genes were extracted and detected. Referring to Luz’s method, total RNA was extracted using the Trizol kit, and each sample was repeated twice [22]. The extracted RNA was screened for quality using Agilent 2100 Bioanalyzer with reference to Riedmaier’s study [23]. The cDNA libraries were sequenced using the Illumina HiSeqTM2500 platform from Guangzhou Kidio Biotechnology Co., Guangzhou, China [24].

Data quality control was performed to filter out low-quality data. UniGene assembly and functional annotation were conducted, and gene expression was quantified. Significantly different genes were screened based on an FDR < 0.05 and |log2FC| > 1 using DESeq v1.20.0 software (Bioconductor-DESeq, Boston, MA, USA). We utilized the KEGG (Kyoto Encyclopedia of Genes and Genomes) and GO (Gene Ontology) databases for functional enrichment analysis of the differential genes. Functional blocks of differential gene enrichment were analyzed to identify core pathways contributing to the differential expression of floral genes. The selection criteria for significant differential genes were |log2FC| > 1 and FDR < 0.05 [25]. GSEA (gene set enrichment analysis) was employed to uncover informative genes with subtle variation, complementing the results of differential analysis and ensuring the comprehensiveness of the findings. Weighted gene coexpression network analysis (WGCNA) was conducted to examine the relationship between gene modules and flower color traits, identifying correlations between flower color and gene modules and screening key pathways and key genes associated with flower color. The analysis involved the use of Fastp 0.18.0, Trinity v2.8.4, Blastx 2.3.5, and DESeq v1.20.0, among others. The differential gene interaction networks were analyzed using STRING: functional protein association networks [26]. The interaction network diagrams were constructed using Cytoscape 3.9.0 [27].

### 2.4. Analysis of Flower Color Regulation

Shared pathway analysis of mined differential metabolites and differentially expressed genes present in abundance using combined transcriptomic–metabolomic analysis [28]. Correlation between screened key genes and key metabolites was assessed using Pearson’s correlation coefficient analysis [29]. Cytoscape 3.9.0 (Cytoscape 3.9.0 Release Notes) was used to map the correlation network, and the database used was the KEGG database (KEGG: Kyoto Encyclopedia of Genes and Genomes).

### 2.5. Key Structural Gene Expression Validation Methods

Firstly, primers were designed using Primer 6 for qRT-PCR validation. The steps were as follows. RNA samples were processed according to the instructions of the HiScript reverse-transcriptase kit. The RNA was initially thawed on ice, and the mixture was prepared and mixed based on the reaction system outlined in Table S1. After a brief centrifugation, it was incubated at 42 °C for 2 min. Subsequently, the mixture was prepared following the reverse-transcription reaction system described in Table S1, thoroughly mixed, and incubated at 50 °C for 50 min. Finally, it was incubated at 85 °C for 5 min to obtain the cDNA. The internal reference gene selected was RhACT [13], and the qRT-PCR reagent (HiScript reverse-transcriptase kit) from HiScript was used to quantify the gene using a fluorescent quantitative PCR instrument (ABI StepOne Plus) according to the appropriate fluorescent quantitative PCR reaction system and PCR reaction conditions. Fluorescent dye selection SYBRGreenI (molecular probe).

Secondly, we employed the gray correlation method to analyze the correlation between each key metabolite and key genes during flowering and identify structural genes with a high correlation with key floral pigments. Gene sequences were converted into protein sequences using MEGA 7 (MEGA 7—Software finder—University of Kent). Subsequently, Basic Local Alignment Search Tool (BLAST) was used to compare homologous sequences and determine significant structural genes.

## 3. Results

### 3.1. Key Metabolite Extraction Results and Analysis

#### 3.1.1. Key Metabolites

**Figure 2:**
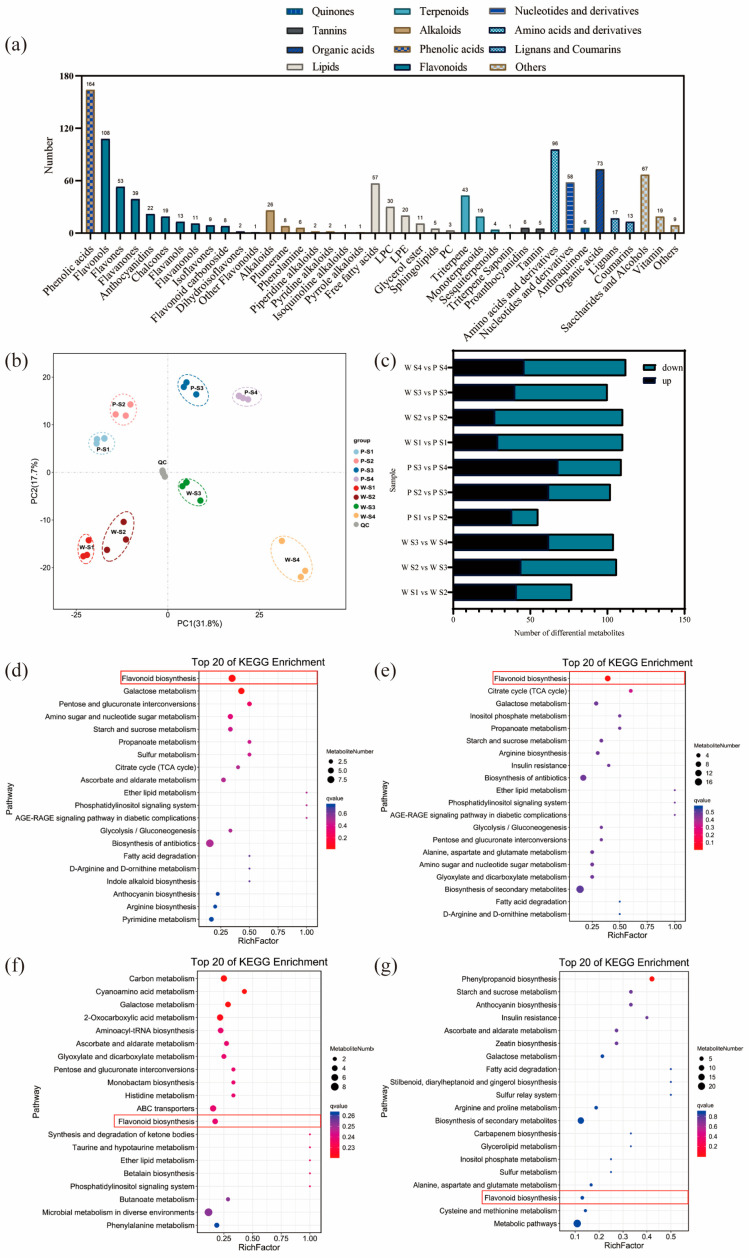
Analysis of metabolites and differential metabolites between periods in *R. latoucheae*. (**a**) a statistical chart of the metabolic classes and numbers contained in the petals of *R. latoucheae*. (**b**) PCA principal component analysis of metabolites in petals in various periods. (**c**) Number of differential metabolites, Screening for differential metabolites based on VIP ≥ 1 and *t*-test *p* < 0.05. (**d**–**g**) KEGG enrichment analysis of differential metabolites. From top to bottom, the enrichment analysis of the differential metabolite KEGG in S1 (**d**), the enrichment analysis in S2 (**e**), the enrichment analysis in S3 (**f**), and the enrichment analysis in S4 (**g**).

The results of the analysis on the differential relationships and related regulatory pathways of metabolites at different developmental stages are presented in Figure 2a. A total of 1057 metabolites were detected in the petals of white-pink and purple plants, with notable compounds including phenolic acids, flavonoids, and amino acids. Based on the primary classification of substances, 11 different types of flavonoid metabolites were detected. In terms of secondary classification, 96 amino acid-derived metabolites (9.082%), 164 phenolic acid metabolites (15.516%), 58 nucleotides and their derivatives (5.487%), and 285 flavonoid metabolites (26.964%) were detected. PCA analysis was conducted on the metabolites from different groups (Figure 2b), and the samples within each group of the two flowering plants showed good replication with significant differences observed between groups. The overall quantity of differential metabolites in the white-pink and purple plants did not vary significantly during flowering, as determined by screening differential metabolites with VIP ≥ 1 and *t*-test *p* < 0.05. However, as the flowers opened, there was a notable increase in differential metabolites, leading to larger differences in flower color (Figure 2c). The enrichment analysis of differential metabolites in the KEGG database resulted in the selection of the top 20 pathways with the smallest Q values for plotting the differential metabolite pathway enrichment. In the graph, a higher value indicates a greater number of metabolic differential metabolites, and a redder color indicates a smaller Q value. During the S1 period, differential metabolites were significantly enriched in pathways such as flavonoid, galactose, pentose and glucuronide conversion, amino and nucleotide sugars, starch and sucrose, and protein and other metabolic pathways. Among them, eight differential metabolites were enriched in the flavonoid metabolic pathway (Figure 2d). In the S2 period, differential metabolites showed significant enrichment in pathways including flavonoid, TCA cycle, galactose, inositol phosphate, propionate, starch and sucrose, arginine, and other metabolite synthesis pathways. Nine differential metabolites were annotated in the flavonoid metabolic pathway (Figure 2e). During the S3 period, the differential metabolites were clustered in carbon metabolism, galactose, histidine, and flavonoid metabolic pathways. Four differential metabolites were significantly enriched in the flavonoid metabolic pathway, and two differential metabolites were significantly enriched in the anthocyanin metabolic pathway (Figure 2f). In the S4 period, differential metabolites were notably enriched in the phenylpropanoid metabolic pathway. Three differential metabolites were identified in the flavonoid synthesis pathway, and another three differential metabolites were found in the anthocyanin metabolic pathway (Figure 2g). Therefore, throughout the flowering process, more differential metabolites were enriched in the flavonoid metabolic synthesis pathway. The differential metabolites in the flavonoid metabolic pathway were closely associated with flower color differences, suggesting that the compounds involved in the flavonoid synthesis pathway warrant further analysis.

#### 3.1.2. Effect of Flavonoid Metabolites on Flower Color

The results of the trend analysis of flavonoid metabolites are depicted in Figures S1 and S2. As shown in the figures, malvidin-3-galactoside, peonidin-3,5-diglucoside, malvidin-3-O-rutinoside, malvidin-3-O-glucoside, and cyanidin-3-O-glucoside exhibited an accumulation pattern during the flowering of purple plants, indicating their key role in the formation of purple flower color. In the S4 period, the purple petals appeared lighter compared to the S3 period, and there was a decreasing or plateauing trend observed for peonidin-3-O-sambubioside and petunidin-3-O-glucoside-5-O-arabinoside, similar to the trend in purple flower color. Metabolites such as flavonols and dihydroflavonols, which are indirectly related to flower color formation, were mainly enriched in module 9 and remained stable during the S1–S3 period, but showed a declining trend in the S4 period. In contrast, in white-pink plants, most of the anthocyanins directly associated with flower color were enriched in module 17 and exhibited an increasing trend followed by a plateau. For instance, cyanidin-3-O-glucoside, peonidin-3-O-glucoside, malvidin-3-O-galactoside, malvidin-3-O-glucoside, peonidin-3-O-sambubioside, peonidin-3,5-O-diglucoside, and malvidin-3-O-rutinoside showed a cessation in their increment after the S2 period, which corresponded to the fading of white-pink flower color after the S2 period. The trends in the content of these metabolites aligned with the changes in flower color during the S3 and S4 periods. Flavonols indirectly related to flower color, such as dihydroflavonol, were primarily enriched in modules 9, 7, and 0, and their trends exhibited a decrease throughout the flowering process. For example, rhododendron xanthin and popcornin began to decline in the S2 period. The trends of naringenin, dihydrokaempferol, and taxifolin in the white-pink petals were more similar to those in purple plants, with the content remaining relatively stable during the S1–S3 period and decreasing sharply during the S4 period. These results indicate that most anthocyanins in purple plants exhibited an increasing trend, while the content of anthocyanins in white-pink plants remained consistent after the period of color change. Furthermore, the differences between white-pink and purple plants were primarily observed during the early flowering and blooming stages following the color change period, which corresponded to the trends observed for anthocyanins such as peonidin-3,5-O-diglucoside, peonidin-3-O-sambubioside, malvidin-3-O-glucoside, malvidin-3-O-galactoside, cyanidin-3-O-glucoside, and malvidin-3-O-rutinoside.

#### 3.1.3. Effect of Anthocyanins on Flower Color

The significant differences of anthocyanins in each period during flowering are presented in Table S2. Among the 22 measured anthocyanins, 10 of them exhibited significant differences. Throughout the flowering process, the content of anthocyanins increased in both colors of petals. However, the rate of increase in anthocyanin content in white-pink petals started to slow down after the early flowering period (Figure S3), leading to differences in the abundance of anthocyanin expression between the early flowering period and the blooming period. In the S1 period, significant differences were observed between the two colors of petals for malvidin-3-O-rutinoside, malvidin-3-O-glucoside, and malvidin-3-O-galactoside. In the S2 period, the petals of the two colors showed significant differences in cyanidin-3,5-dio-O-glucoside. In the S3 and S4 periods, malvidin-3-O-glucoside, malvidin-3-O-galactoside, malvidin-3-O-rutinoside, peonidin-3-O-glucoside, and cyanidin-3-O-glucoside in the purple petals were significantly higher compared to those in the white-pink petals.

**Figure 3:**
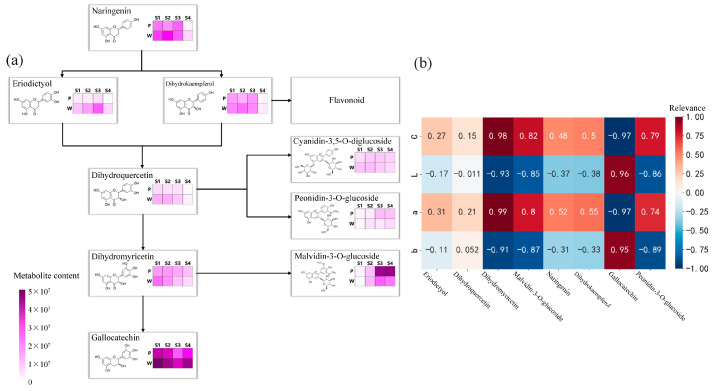
Determination of anthocyanin pigment synthesis pathways and metabolite correlation analysis in *R. latoucheae*. (**a**) Diagram of anthocyanin synthesis network of *R. latoucheae*. (**b**) Heat map of correlation between key metabolites and flower color. Red indicates positive correlation, blue indicates negative correlation, the higher the correlation the darker the color. Note: P: purple; W: white; b: yellowish blue coloration; a: red-green coloration; L: degree of lightness; C: color level.

The results of the analysis of the anthocyanin synthesis mode are presented in Figure 3a. During flowering, the contents of metabolites such as naringenin, eriodictyol, dihydrokaempferol, dihydroquercetin, dihydromyricetin, peonidin-3-O-glucoside, malvidin-3-O-glucoside, and gallocatechin exhibited differences, with the most significant differences observed in the content of malvidin-3-O-glucoside and gallocatechin in the dihydromyricetin branch. The anthocyanin that exhibited the most significant difference in purple plants during the S3 and S4 periods was malvidin-3-O-glucoside. Finally, the correlation analysis (Figure 3b) between the content of differential metabolites and flower color during the S3 and S4 periods revealed weak correlations between metabolites such as naringenin, eriodictyol, dihydrokaempferol, and dihydroquercetin, and flower color presentation. Dihydromyricetin, malvidin-3-O-glucoside, and peonidin-3-O-glucoside showed significant positive correlations with the C and a values of antler *Rhododendron* color. Gallocatechin exhibited a negative correlation with the C and a values of flower color. Therefore, dihydromyricetin, malvidin-3-O-glucoside, malvidin-3-O-galactoside, gallocatechin, and peonidin-3-O-glucoside are the key floral pigments that influence flower color during the early flowering stage and in full bloom.

### 3.2. Key Gene and Key Pathway Assay Results and Analysis

By conducting sequencing quality control, a total of 1,710,291,924 clean reads were obtained from all samples, which were subsequently aligned with gene sequences from four essential public databases, Nr, KOG, KEGG, and Swiss-Prot. The assay exhibited an average error rate of less than 3%, with average Q20 and Q30 values of 97% and 94%, respectively. The GC content ranged from 46.52% to 47.92% (Table S3). The base composition was well-balanced, indicating high-quality sequencing and data filtering, and the data were deemed suitable for subsequent analysis. The clean data were assembled by splicing through Trinity software (v2.8.4), resulting in an average length of the assembled Unigene N50 of 1390 bp (Figure S4). The correlation coefficients of the three replicate samples for each period were all greater than 0.85 (Figure S5), indicating good intra-group reproducibility for the subsequent study.

**Figure 4:**
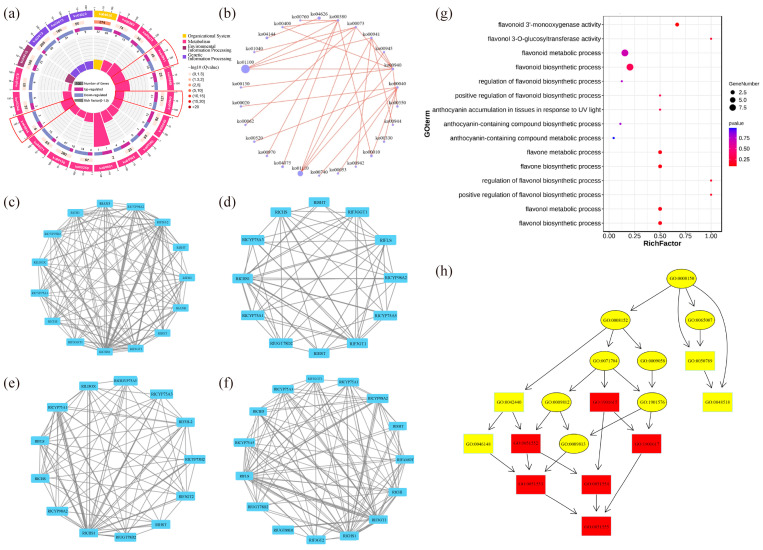
Functional enrichment by GO and KEGG databases to identify differential genes in *R. latoucheae*. (**a**) Differential gene enrichment circle map, the first circle with different colors indicates the KEGG primary pathway; the second circle shows the number of this pathway in the background genes; the third circle shows the up- and downregulation of differential genes enriched in this pathway; the fourth circle: the rich factor value of each pathway. (**b**) Differential gene pathway interaction network. (**c**–**f**) Differential unigene interaction networks at different periods, from top to bottom, the unigene interaction networks in S1 (**c**), the unigene interaction networks in S2 (**d**), the unigene interaction networks in S3 (**e**), and the unigene interaction networks in S4 (**f**). (**g**) GO enrichment of anthocyanin and flavonoid-related unigene. (**h**) GO-directed acyclic of anthocyanin and flavonoid synthesis-related unigene.

The top 20 pathways that exhibited significant enrichment, as determined by FDR values, were selected and visualized in an enrichment circle diagram (Figure 4a). Pathways such as flavonoid synthesis (Ko00941), phenylpropane synthesis (Ko00940), flavonoid and flavonol synthesis (Ko00944), and flower color-related pathways were significantly enriched in white-pink and purple plants, suggesting their potential involvement in flower color formation. Based on the pathway information associated with the differential genes, a core pathway network diagram of the differential genes was generated (Figure 4b), illustrating the interactions between pathways in the KEGG database. Among these core pathways, phenylpropane synthesis (Ko00940) emerged as the central pathway, with eight pathways connected to it. This pathway influenced anthocyanin (Ko00942) and flavonoid and flavonol (Ko00944) synthesis by regulating flavonoid synthesis (Ko00941). Notably, genes related to anthocyanin glycoside synthesis, such as Unigene0010070 (F3GT1) and Unigene0047366 (F3GT1), exhibited higher expression levels in purple plants compared to white-pink plants.

The results of the analysis of differential gene interaction networks from the STRING Interaction Network Database are presented in Figure 4c–f. In the S1 stage, *RlCHS1*, *RlF3GT1*, and *RlF3H-2* (Unigene0045101, Unigene0046184) exhibited strong correlations with other differential genes (Figure 4c). In the S2 stage, *RlCHS1* (Unigene0053893, Unigene0053894), *RlF3GT1* (Unigene0010070, Unigene0040485, Unigene0047366) showed high correlation with other differential genes (Figure 4d). Similarly, in the S3 stage, *RlCHS1* (Unigene0053893, Unigene0053894), *RlCYP75A1* (Unigene0046184, Unigene0047773), *RlUGT78D2* (Unigene0001660) displayed strong correlations with other differential genes (Figure 4e). In the S4 stage, *RlCHS1* (Unigene0053893, Unigene0053894), *RlF3GT1* (Unigene0010070, Unigene0040485, Unigene0047366) exhibited high correlation with other differential genes (Figure 4f). These findings highlight the significant connectivity of *RlCHS1* and *RlF3GT1* with other differential genes during flowering.

The enrichment of differential genes in 15 terms related to anthocyanin and flavonoid synthesis, as determined by GO secondary classification analysis, is illustrated in Figure 4g. The directed acyclic graph of the GO pathway indicates that “flavonoid biosynthesis process” and “flavonol metabolism process” are crucial nodes in the differential GO pathway (Figure 4h). Although white-pink and purple plants were enriched in these terms, the enrichment was not statistically significant. The differential genes were sorted and de-duplicated, and their log2 (FC) values demonstrated the magnitude of the differences (Table S4). *RlMYB12* (Unigene0023540), *RlRHM1* (Unigene0048513), and RlOs02g0190300 (Unigene0036583) exhibited higher abundance in the petals, while *RlCHS* (Unigene0047699), *RlCHS1* (Unigene0037903), and *RlF3H-2* (Unigene0045101) were more prominently expressed. These genes thus played a significant role in the formation of floral color differences.

**Figure 5:**
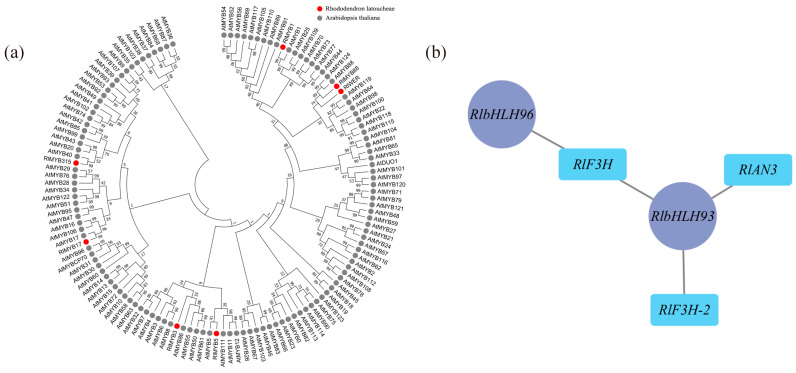
Determination of flower color in relation to key transcription factors using WGCNA. (**a**) Evolutionary tree analysis of key transcription factors in the maroon module and sequences containing the R2R3 structural domain in Arabidopsis, red circles represent transcription factors of *R. latoucheae* and gray circles represent transcription factors of *Arabidopsis thaliana*. (**b**) Transcription factors and structural gene interactions in maroon, *RlbHLH93* and *RlbHLH 96* have regulatory relationships with flavonoid biosynthesis-related structural genes *RlF3H*, *RlF3H-2*, and *RlANS* in the maroon module.

According to the weighted gene coexpression network analysis (WGCNA), 18 modules were identified (Figure S6). Among these modules, the maroon module exhibited the highest number of enriched genes, totaling 4548. Correlation analysis was conducted using the phenotypic data of module eigenvalues and flower color (Table S5), revealing that the maroon module displayed the strongest correlation with flower color. The metabolic pathways and genes associated with flavonoid metabolite synthesis in the maroon module were compiled and analyzed, resulting in the identification of 18 differential genes enriched in Ko00941 (Table S6), which showed significant correlations with flower color b-values and color intensity. Transcription factors are known to play a crucial role in transcriptional regulation, and within the maroon module, there were 236 transcription factors, including 22 MYB family transcription factors and 21 bHLH family transcription factors. Most MYB proteins associated with plant floral color accumulation belong to the R2R3-MYB protein group. To further investigate the transcription factors associated with floral color, the MYB family transcription factors enriched in this module underwent sequence analysis, using amino acid sequences containing R2R3 structural domains in Arabidopsis as references. A comparison was made between the 22 MYB family sequences in the maroon module and those containing R2R3 structural domains in Arabidopsis. As a result, a total of 7 transcription factors were identified as R2R3-MYB transcription factors, and a gene family evolutionary tree analysis was conducted on the sequences containing R2R3-MYB transcription factors in Arabidopsis (Figure 5a). As shown in the figure, three out of the seven differential R2R3-MYB transcription factor proteins clustered well with Arabidopsis R2R3-MYB transcription factors: RlMYB1 clustered with Arabidopsis AtMYB1, RlMYB315 clustered with Arabidopsis AtMYB40, and RlMYB17 clustered with Arabidopsis *AtMYB17*. Through reciprocal network analysis (Figure 5b), it was observed that the transcription factors RlbHLH93 and RlbHLH96 were regulated along with the flavonoid biosynthesis-related structural genes *RlF3H, RlF3H-2*, and *RlANS* in the maroon module.

### 3.3. Metabolite Regulation Analysis

**Figure 6:**
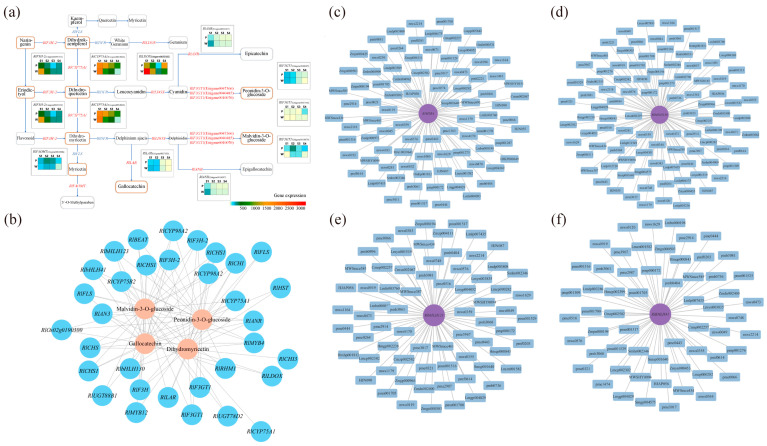
Probing the relationship between key genes and key metabolites using comprehensive analysis. (**a**) Network prediction of anthocyanin synthesis in *R. latoucheae* based on KEGG database. The rounded rectangles indicate metabolites, yellow indicates different metabolites, the right-angled rectangle indicates structural genes, and the red letter indicates that the expression of the gene is different in four periods. (**b**) Network diagram of the correlation between gene expression and metabolite content. Where blue circles indicate genes, pink circles indicate metabolites, and lines indicate correlations. (**c**–**f**) Analysis of transcription factor *RlMYB4*, *RlbHLH130*, *RlbHLH123*, *RlbHLH41* and differential metabolite interaction networks, differential metabolites with correlation coefficients ≥ 0.8 and *p* ≤ 0.05 with *RlMYB4*, *RlbHLH130*, *RlbHLH41*, and *RlbHLH123* transcription factors were selected for gene and metabolite interaction network analysis.

Through the previous differential screening and differential enrichment analysis, the KEGG pathway information common to the differential genes and differential metabolites was obtained, so the KEGG network diagram of anthocyanin synthesis was predicted (Figure 6a). As can be seen from the figure, *RlLDOX* (Unigene0033582), *RlLAR* (Unigene0027133), *RlANR* (Unigene0016387), *RlF3GT1* (Unigene0010070, Unigene0040485, Unigene0047366), *RlF3H-2* (Unigene0045101), *RlCYP75A1* (Unigene0012061), *RlFAOMT* (Unigene0026806) and other differential structural genes are involved in the synthesis of anthocyanins and flavonols.

The results of the transcription factor correlation analysis for five differential metabolite-related MYB family and bHLH family members, including dihydromyricetin, malvidin-3-O-glucoside, malvidin-3-O-galactoside, gallocatechin, and peonidin-3-O-glucoside, are presented in Table S7. The analysis revealed significant correlations of *RlMYB4* (Unigene0035230), *RlbHLH130* (Unigene0051207), *RlbHLH41* (Unigene0047378), and *RlbHLH123* (Unigene0024345) with key metabolites. Specifically, *RlbHLH41* displayed a significant positive correlation with malvidin-3-O-glucoside and peonidin-3-O-glucoside, while *RlbHLH123* exhibited a significant positive correlation with gallocatechin and a significant negative correlation with malvidin-3-O-glucoside and peonidin-3-O-glucoside. Apart from *RlMYB4*, *RlbHLH130*, *RlbHLH41*, and *RlbHLH123*, other transcription factors, such as *RlMYB12* (Unigene0023540), *RlMYB1* (Unigene0020648), *RlMYB17* (Unigene0031302), *RlMYB315*, *RlbHLH93* (Unigene0009443), and *RlbHLH96* (Unigene0029233), showed correlations with certain dihydroflavonols and flavonoid substances, including anthocyanins. However, these correlations were not significant for metabolites such as dihydromyricetin, malvidin-3-O-glucoside, malvidin-3-O-galactoside, gallocatechin, and peonidin-3-O-glucoside (Table S8). Therefore, *RlMYB12*, *RlMYB1*, *RlMYB17*, *RlMYB315*, *RlbHLH93*, and *RlbHLH96* are involved in the regulation of flower color but are not key transcription factors for purple flower color.

The results of the gene and metabolite interaction network analysis for key transcription factors and differential metabolites are depicted in Figure 6c–f. The significant differential metabolites associated with *RlMYB4* were primarily sugars, alcohols, anthocyanins, and flavonols. *RlbHLH130* exhibited significant associations with anthocyanins, flavonoids, and triterpenoids. *RlbHLH41* showed significant associations with anthocyanins, triterpenoids, phenolic acids, and flavonoids. Furthermore, the significant differential metabolites associated with *RlbHLH123* (Unigene0024345) included triterpenes, anthocyanins, organic acids, and flavanols. Hence, *RlMYB4*, *RlbHLH130*, *RlbHLH41*, and *RlbHLH123* are all involved in anthocyanin and flavonoid metabolism.

Correlation analysis was conducted based on the differential genes and differential metabolites associated with flower color, and the correlation network diagram illustrating gene expression and metabolite abundance is presented in Figure 6b. The differential genes strongly correlated with dihydromyricetin were *RlAN3* (Unigene0046185), *RlCHI* (Unigene0043745), *RlCYP75A1* (Unigene0012061), and *RlF3H* (Unigene0045102). For genes related to malvidin-3-O-glucoside and peonidin-3-O-glucoside, *RlbHLH130* (Unigene0051207), *RlbHLH41* (Unigene0047378), *RlbHLH123* (Unigene0024345), *RlMYB4* (Unigene0035230), and *RlF3GT1* (Unigene0010070, Unigene0040485, Unigene0047366, Unigene0047369) displayed strong associations. Lastly, the differential gene strongly associated with gallocatechin was *RlbHLH123* (Unigene0024345).

### 3.4. Validation Analysis of Expression of Key Structural Genes

**Figure 7:**
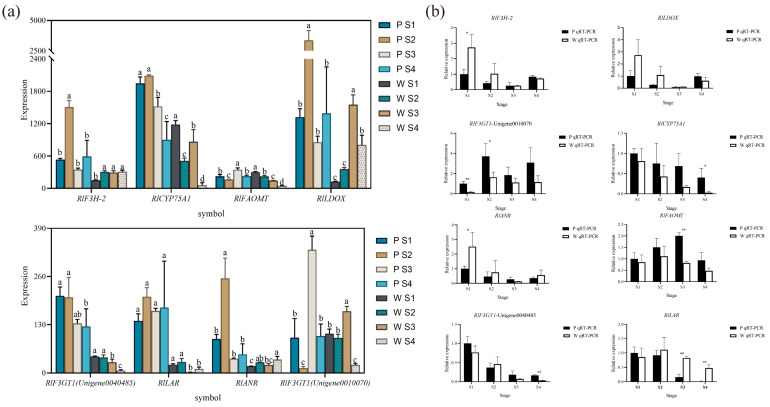
qRT-PCR experiments to verify the reliability of key structural gene expression level. (**a**) Differential gene expression map related to flower color, different letters (a–d) indicate significant differences in the expression levels of differentially expressed genes during the flowering process of *Rhododendron* antlers (*p* < 0.05) (**b**) qRT-PCR to verify the expression of *RlANR*, *RlFAOMT*, *RlF3GT1* (Unigene0040485), *RlLAR*, note: * indicates *p* < 0.05, ** indicates *p* < 0.01 (*p* vs. w), the error bars in the graph represent the standard error.

Based on the combined metabolomic, transcriptomic, and two-omics analyses, eight differential genes associated with flower color (Figure 7a) were initially screened, exhibiting significant differences in expression. According to the qRT-PCR expression results, *RlCYP75A1*, *RlFAOMT*, *RlF3GT1*, *RlLAR*, *RlANR*, *RlF3H-2*, and *RlLDOX* were found to be differentially expressed during flowering in both white-pink and purple plants (Figure 7b). Specifically, significant differences were observed in the expression of *RlF3GT1* during the S2 stage, *RlLAR* and *RlFAOMT* during the S3 period, and *RlCYP75A1* and *RlLAR* during the S4 stage.

The mean expression of *RlCYP75A*1 and *RlFAOMT* consistently exhibited higher levels in purple plants compared to white-pink plants during flowering. The anthocyanin-3-O-glucosyltransferase gene *RlF3GT1* (Unigene0010070, Unigene0040485) displayed higher expression in purple plants than in white-pink plants during the S3 and S4 stage. *RlLAR* (Unigene0027133) showed higher expression in white-pink plants from the S2 to S4 stage, resulting in the production of more colorless gallocatechin in white-pink plants. *RlANR* (Unigene0016387) converts colored anthocyanins to colorless epicatechin and epigallocatechin, and its expression was higher in white-pink plants during the S1, S2, and S4 stage. *RlF3H-2* (Unigene0045101) exhibited higher expression in white-pink plants during the S1 and S2 stage, while its expression in the petals of white-pink and purple plants was essentially the same during the S3 and S4 stage. The content of *RlLDOX* (Unigene0033582) was higher in white-pink plants than in purple plants during the S1 and S2 stage, and higher in purple plants during the S4 stage.

## 4. Discussion

### 4.1. Differences in White-Pink and Purple Flower Color Pathways

In our study, we observed differences in photosynthesis and flavonoid synthesis pathways between white-pink and purple *R. latoucheae* plants. However, the key factor contributing to the change in flower color was found to be the differences in flavonoid metabolite synthesis pathways. Metabolomic analysis revealed that the main distinctions between white-pink and purple plants were observed in the metabolic pathways of flavonoids and anthocyanins, and the content of these compounds showed significant correlations from flower color. Transcriptomic analysis also identified differences in the flavonoid synthesis pathway, flavonoid and flavonol synthesis pathway, anthocyanin 3-O-glucosyltransferase GO terms, and flavonol 3-O-glucosyltransferase activity GO terms between white-pink and purple plants. These pathways are all involved in the synthesis of flavonoids, anthocyanins, and flavonols. These findings support the notion that there are substantial differences in flavonoid metabolism between pink and purple plants, which aligns with the inference made by Du et al., who found that anthocyanins and flavonols are crucial pigments for flower color in most *Rhododendronaceae* species [12]. Differences in flavonoid, flavonol and anthocyanin synthesis pathways can have an impact on flower color presentation [30]. Li et al. used histological analysis to uncover that flavonoid metabolic pathways are associated with buckwheat flowers color [31], Tyrach et al. found that flower color in Gerbera was associated with differential genes in the flavonoid synthesis pathway [32]. These studies again support that differential genes enriched in the flavonoid pathway in white-pink and purple *R. latoucheae* are responsible for the differences in white-pink and purple flower color.

Although KEGG enrichment analysis is commonly used to study differences in plant floral color [33], it may overlook metabolites with low expression but significant relevance. In contrast, GSEA analysis can leverage all genes and explore informative genes with subtle variations [34]. In our study, GSEA analysis revealed that white-pink and purple plants differed in metabolic pathways related to photosynthesis, such as chlorophyll, photosynthesis-antenna protein, and other pathways, which exhibited significant positive correlations with purple plants. This indicates that the differences between white-pink and purple plants extended beyond the pathways associated with flavonoid and anthocyanin synthesis to include pathways related to photosynthesis. Notably, these photosynthesis-related pathways were not found to be associated with the anthocyanin synthesis pathway in the reciprocal network analysis.

### 4.2. Key Metabolites Regulating Purple Flower Color

In our study, we observed that the purple flower color in *R. latoucheae* was influenced by the dihydromyricetin branch of the flavonoid metabolic pathway. Differences in the content of malvidin-3-O-glucoside and peonidin-3-O-glucoside contributed to the variations in flower color between white-pink and purple plants. An increase in the content of malvidin-3-O-glucoside and peonidin-3-O-glucoside, along with a decrease in gallocatechin content, resulted in a purple flower color. UPLC-MS/MS analysis revealed that the petals contained higher levels of flavonoid metabolites. Furthermore, the flower color of white-pink plants started to fade after the S2 period, whereas the accumulation of anthocyanosides associated with flower color was observed in purple plants during the flowering process. Numerous studies have demonstrated the association between purple flower color and flavonoid metabolites. Du et al. investigated flower color and anthocyanin content in seven subgenera of *Rhododendron* and found that anthocyanins, particularly mallow pigments and delphinidin, contribute to the purple color of petals [12]. Thus, it was hypothesized that the difference in flower color between white-pink and purple plants is primarily due to the presence of anthocyanins in flavonoid compounds.

Wang et al. identified mallowin as the key floral pigment in the petals of purple *Rhododendron Pulchrum Sweet*, and an increased content of malvidin-3-O-glucoside resulted in a purple-red color [13]. Liu et al. also found that cyanidin was the primary chromogenic metabolite in the red-purple *Rhododendron triflorum*, while malvidin chloride was the main pigment in purple rhododendrons such as *Rhododendron nivale* and *Rhododendron oreotrephes* [35]. These studies align with our findings, which suggest that the main differences between white-pink and purple colors are concentrated in the dihydromyricetin branch, and that dihydromyricetin, gallocatechin, malvidin-3-O-glucoside, and peonidin-3-O-glucoside are significantly associated with flower color. The impact of malvidin chloride on purple flower color is not limited to *Rhododendronaceae* but extends to other plants, where the formation of purple color is correlated with malvidin chloride and other anthocyanins, with the highest content found in *Solanum lycopersicum* L. [36].

### 4.3. Structural Genes Regulating Differences in Purple Flower Color

Our study revealed that *RlF3GT1* (Unigene0010070, Unigene0040485, Unigene0047366), *RlLAR* (Unigene0027133), and *RlANR* (Unigene0016387) are key structural genes affecting the flower color of purple *R. latoucheae*.

Differential genes in the flavonoid metabolic pathways were found to be strongly correlated with flower color traits in the cDNA library through Illumina sequencing. Forty-five differential genes were enriched in the flavonoid, anthocyanin, flavonoid, and flavonol metabolic pathways during flowering in white-pink and purple plants. Two sets of joint analyses revealed a significant enrichment of differential genes and differential metabolites in the flavonoid, anthocyanin, flavonoid, and flavonol synthesis pathways. Differential structural genes such as *RlLDOX* (Unigene0033582), *RlLAR* (Unigene0027133), *RlANR* (Unigene0016387), *RlF3GT1* (Unigene0010070, Unigene0040485, Unigene0047366), *RlF3H-2* (Unigene0045101), *RlCYP75A1* (Unigene0012061), and *RlFAOMT* (Unigene0026806) are involved in the synthesis of malvidin-3-O-glucoside, peonidin-3-O-glucoside, gallocatechin, and dihydromyricetin. Key structural genes in the anthocyanin synthesis pathway, such as *lDOX*, *LAR*, *ANR*, *F3GT1*, *F3H-2*, *CYP75A1*, and *FAOMT*, have been reported in numerous articles related to flower color. For example, Yang et al. discovered a negative correlation between the expression of *ANR* and color in *Pyrus communis* L. [37]; Wu et al. found that DNA methylation of *LDOX* genes contributed to peach flower color variation [38], and Dai et al. revealed an association between the expression of *F3H* and anthocyanin/flavonoid biosynthesis in mulberry [39]. These findings indicate that the expression of *RlLDOX*, *RlF3GT1*, *RlF3H-2*, and *RlCYP75A1* is positively correlated with anthocyanin content, while the expression of *RlLAR* and *RlANR* is negatively correlated with anthocyanin content.

The cyanidin synthesis regulatory network diagram revealed that RlF3GT1 directly participates in the synthesis of malvidin-3-O-glucoside and peonidin-3-O-glucoside. *RlLAR* is associated with the synthesis of gallocatechin, *RlANR* is involved in the synthesis of gallocatechin and epicatechin, and *RlF3H-2* is associated with the synthesis of metabolites such as dihydromyricetin and dihydroquercetin. The results of the qRT-PCR assay demonstrated a high correlation between *RlF3GT1* and *RlFAOMT* and key anthocyanins.

Morita et al. found that the petunia *3GT* gene can significantly affect its flower color [40]. While *RlLAR* (Unigene0027133) exhibited higher expression levels in white-pink plants from the trans-color stage to the bloom stage, *RlANR* (Unigene0016387) displayed higher expression levels in white-pink plants during the all-green, trans-color, and bloom stages. The colorless anthocyanin reductase *JgLAR* and anthocyanin reductase *JgANR* convert proanthocyanidins into colorless catechins, specifically epicatechin [41], and their content is negatively correlated with the content of anthocyanins [42]. In Theobroma cacao, the levels of several anthocyanin-related genes (*LAR* and *ANR*) consistently differed between green and purple varieties of some Theobroma cacao, and these genes may produce more anthocyanins in purple varieties than in green varieties [43]. The same is also true for grapes [44], peach [45], raspberry [46], tea [47], and begonia [48]. These studies indicate that *RlF3GT1*, *RlLAR*, and *RlANR* are involved in flower color formation.

### 4.4. Key Transcription Factors Regulating Purple Flower Color Differences

Our study discovered that RlMYB1, RlMYB3, and RlMYB12 are involved in regulating flower color in *R. latoucheae* but are not key transcription factors for purple flower color formation. On the other hand, RlbHLH130, RlbHLH41, RlbHLH123, and RlMYB4 are associated with purple flower color formation. The R2R3-MYB protein, the most widely involved transcription factor in plant anthocyanin biosynthesis, can activate the expression of one or more structural genes, thereby promoting anthocyanin synthesis and giving plants a red or purple appearance [49]. The transcription factor RlMYB12 (Unigene0023540) is enriched in flavonoid metabolism, and its expression is significantly higher in purple petals than in white-pink ones. Amino acid sequence analysis revealed that the RlMYB12 protein sequence contains the R2R3 structural domain, consistent with the findings of Yamagi et al. that the LhMYB6 and LhMYB12 transcription factors, isolated from purple Asian hybrid lilies, activate anthocyanin synthesis [50]. This suggests that RlMYB12 may be involved in anthocyanin regulation. However, our study found that RlMYB12 was associated with the synthesis of certain flavonoids and anthocyanins through the two-omics joint analysis, but not with key metabolites such as malvidin-3-glucoside, leading to the speculation that it is not a key transcription factor for purple flower color formation.

WGCNA analysis identified RlMYB315, RlMYB1, and RlMYB17 as being associated with flower color. However, the two-omics joint analysis revealed that RlMYB1, RlMYB3, and RlMYB12 are associated with the synthesis of some flavonoids and anthocyanins and are involved in flower color regulation but are not key transcription factors for purple flower color formation. In contrast, RlbHLH130, RlbHLH41, RlbHLH123, and RlMYB4 showed significant correlations with the content of malvidin-3-O-glucoside, peonidin-3-O-glucoside, gallocatechin, and dihydromyricetin, indicating their potential involvement in regulating purple flower color. The regulatory roles of these transcription factors on flavonoid metabolites have been demonstrated in Arabidopsis, grapefruit, and cauliflower [51,52,53].

### 4.5. Key Genes and Key Metabolite Regulatory Relationships

Our study uncovered a regulatory relationship between the transcription factor RlMYB4, the structural gene *RlLAR*, and the metabolites malvidin-3-O-glucoside and peonidin-3-O-glucoside. RlF3GT1 regulates the synthesis of more malvidin-3-O-glucoside and peonidin-3-O-glucoside through anthocyanin-3-O-glucosyltransferase.

Structural genes can influence flower color by affecting metabolites in the anthocyanin and flavonoid metabolic pathways [54]. *RlF3GT1*, *RlLAR*, and *RlANR* are key structural genes for purple petals, while RlbHLH130, RlbHLH41, RlbHLH12, and RlMYB4 are key transcription factors for purple petals. Malvidin-3-O-glucoside and peonidin-3-O-glucoside are the key anthocyanins that impact purple flower color. Through omics analysis, we hypothesized that increased expression of RlF3GT1, a regulatory gene influencing purple flower color, and decreased expression of *RlLAR* and *RlANR*, which contribute to the synthesis of more malvidin-3-O-glucoside and peonidin-3-O-glucoside, are the key factors behind the purple petal phenotype.

At the same time, the expression level of the transcription factor itself can have a regulatory effect on the expression intensity of structural genes [55]. In the present study, *RlMYB4* was found to be homologous to FaMYB1, a typical anthocyanin repressor transcription factor, and it has been shown that the FaMYB1 transcription factor inhibits the accumulation of anthocyanins and flavonols in tobacco [56]. Ariel Salvatierra et al. found that *FcMYB1* expression decreased when *FcANS* expression increased, while the expression of *FcANR* and *FcLAR* decreased with the decrease in *FcMYB1* [57]. The Figure S5 shows that there is a significant positive correlation between the expression of RlMYB4 and *RlLAR*, and that an increase in the expression of *RlMYB4* and *RlLAR* is followed by a decrease in anthocyanin content, all of which indicate that *RlMYB4* and *RlLAR* act as a repressor of anthocyanins. From Figure S7, it can be observed that RlMYB4 and RlbHLH123 have similar regulation patterns, while RlbHLH41 and RlbHLH130 exhibit similar patterns. This study found that RlMYB4 and RlbHLH123 were negatively correlated with malvidin-3-O-glucoside and peonidin-3-O-glucoside, and RlbHLH41 and RlbHLH130 were negatively correlated with malvidin-3-O-glucoside and peonidin-3-O-glucoside. Therefore, it is hypothesized that RlMYB4 and RlbHLH123 reduce the anthocyanin content of white-pink plants by regulating the expression of *RlLAR*, resulting in a lighter color. There is substrate competition between *RlLAR* and F3GT1, so white-pink plants produce gallocatechin along with a decrease in malvidin-3-O-glucoside and peonidin-3-O-glucoside, which in turn distinguishes them from purple flower color. However, the RlbHLH123 protein was able to cluster with AtbHLH012 of the Arabidopsis subfamily IIIf with a more similar sequence, which is consistent with the finding of Heim et al. that AtbHLH012 was positively correlated with anthocyanins in Arabidopsis [58], but inconsistent with the finding of the present study that RlbHLH123 was negatively correlated with malvidin-3-O-glucoside. It is speculated that the difference may be caused by the co-expression of RlMYB4 and RlbHLH123.

### 4.6. Implications and Further Research

The difference in flower color between white-pink and purple plants is mainly influenced by the content of flower pigments in petals, and understanding the key influencing factors can provide a theoretical basis for flower color regulation and genetic modification in *R. latoucheae*. Philippe et al. found that *AtPAP1* was introduced in purple grapes, and it could interact with *AOMT* in grapes to produce more malvidin chloride [59]. In this study, we also observed higher levels of malvidin-3-O-glucoside and peonidin-3-O-glucoside, as well as increased *RlFAOMT* expression, in purple *R. latoucheae*. Therefore, by introducing *AtPAP1* in combination with *RlFAOMT*, we may be able to produce more malvidin-3-O-glucoside for obtaining more purple flowers in future breeding efforts. Kadomura-Ishikawa et al. found that overexpression of the *FaMYB1* gene in strawberries suppressed flower color [60], In the present study, we also discovered that *RlMYB4* was homologous to *FaMYB1*, and its expression was negatively correlated with malvidin-3-O-glucoside, *RlLAR*, and *RlANR*. By suppressing the expression of the transcription factor *RlMYB4*, we could potentially suppress the expression of *RlLAR* and *RlANR* in white-pink *R. latoucheae* to enhance flower color.

The formation of flower color involves complex mechanisms. In this study, we investigated the factors contributing to flower color formation in purple *R. latoucheae* through total anthocyanin and key genes analysis. We identified key genes associated with anthocyanin synthesis and explored the regulatory mechanism of flower color. Further investigation can be conducted in the future to explore gene expression regulation networks, epigenetic regulation, and the pH value of plant cell vesicles, which are upstream and downstream factors in the regulation of flower color, as well as further functional validation of the screened genes, so as to gain a deeper understanding of the mechanism underlying the presentation of purple flower color.

## 5. Conclusions

In this study, we chose the petals of white-pink and purple *R. latoucheae* for the determination of floral pigment content, transcriptomics and metabolomics analysis. The main conclusions of the study were as follows.

White-pink and purple *R. latoucheae* differ in flower color traits and *R. latoucheae* petals contain flavonoids, anthocyanins and carotenoids. Structural genes such as *RlHST*, *RlFAOMT*, *RlCHS*, *RlCHI*, *RlCYP75A*, *RlLDOX*, *RlLAR*, *RlANR*, *RlF3H*, *RlFLS*, *RlF3GT1* are involved in the synthesis of flavonoids. Anthocyanin-3-O-glucosyltransferase *F3GT1*, proanthocyanidin reductase *LAR*, and anthocyanin reductase *ANR* are the key structural genes affecting flower color. RlMYB4, RlbHLH130, RlbHLH41, and RlbHLH123 are the key transcription factors affecting flower color.

*RlF3GT1* affects the flower color of *R. latoucheae* by influencing the synthesis of malvidin-3-O-glucoside and peonidin-3-O-glucoside. *LAR* converts white delphinine to colorless gallocatechin, which forms more gallocatechin in white-pink plants during early and full bloom, resulting in lighter flower color. *ANR* reduces colored anthocyanins to colorless epicatechin and epigallocatechin, which leads to lighter white and pink flowers than purple flowers. *RlMYB4* was negatively correlated with malvidin-3-O-glucoside and peonidin-3-O-glucoside and positively correlated with the structural gene *RlLAR*, and *RlMYB4* could influence the expression of malvidin-3-O-glucoside and peonidin-3-O-glucoside by regulating the expression of the proanthocyanidin reductase gene *LAR*.

In conclusion, it is speculated that the increase in *RlF3GT1* expression and the decrease in *RlLAR* and *RlANR* expression can regulate the synthesis of more malvidin-3-0-glucoside and peonidin-3-O-glucoside in *R. latoucheae*, which leads to the purple color of flowers. There is a regulatory relationship between the transcription factor RlMYB4, the structural gene RlLAR, and the metabolites malvidin-3-0-glucoside and peonidin-3-O-glucoside. Therefore, it is possible to make the flowers more colorful by suppressing the expression of *RlLAR* and *RlANR* in white-pink antler *rhododendron* by suppressing the expression of transcription factor RlMYB4.

## Data Availability

Not applicable.

## Supplementary Materials

The following supporting information can be downloaded at: https://www.mdpi.com/article/10.3390/plants12162897/s1, Figure S1: Trends of Flavonoid Differential Metabolites in Purple Rhododendron latoucheae; Figure S2: Trends of Flavonoid Differential Metabolites in White-pink Rhododendron latoucheae; Figure S3: Anthocyanin content in Rhododendron latoucheae; Figure S4: Unigene Length Distribution; Figure S5: Heat Map of Correlation for Each Sample. The FPKM values were used to indicate the expression of each gene of R. latoucheae. And by calculating the pearson correlation coefficient of FPKM values of the samples, the correlation heat map of the samples was plotted; Figure S6: Module Level Clustering Diagram; Figure S7: Heat map of Correlation between Key Structural Genes and Key Transcription Factors. note: * indicates *p* < 0.05, ** indicates *p* < 0.01, *** indicates *p* < 0.001; Table S1: Reaction conditions required for qRT-PCR experiments; Table S2: Vip and Log2 (FC) statistics of Different Anthocyanins of Rhododendron latoucheae; Table S3: Composition of Bases and their Quality Values; Table S4: Anthocyanin and Flavonoid Synthesis-related Unigene Log2 (FC) values; Table S5: Heat Map of Correlation between Floral Traits and Each Module; Table S6: Unigene Related to Flavonoid in the Maroon Module; Table S7. The Correlation of MYB1, MYB3, MYB4, MYB12 and Key Metabolites; Table S8. The Transcription Factors RlMYB12, RlMYB1, RlMYB17, RlMYB315, RlbHLH93 and RlbHLH96 are Correlated with Key Metabolites.
