# Supplementary material for: Insight into the Molecular Mechanism of Flower Color Regulation in Rhododendron latoucheae Franch: A Multi-Omics Approach
Source: Plants (Basel). 2023 Aug 8;12(16):2897. doi: 10.3390/plants12162897 (PMC10458524; doi:10.3390/plants12162897)
Supplement: Supplementary file 1 [file plants-12-02897-s001.zip › plants-2489531-supplementary.pdf]

plants

Insight into the molecular mechanism of flower color regulation in *Rhododendron latoucheae.*

1. Materials and Methods

Table S1. Reaction conditions required for qRT-PCR experiments.

Reaction step	Name of reactants	Dose of reactants	Reaction temperature	Reaction time	Number of cycles
**DNA removal reaction system**	4×gDNA wiper Mix	4 µl			
	Total RNA	≤2ug			
	RNase-Free ddH2O	16 µl			
**Reverse transcription reaction system**	Previous step Reaction solution	16 µl			
	5×HiScript	4 µl			
**Fluorescent quantitative PCR reaction system**	2 x ChamQ SYBR qPCR Master Mix	10 µl			
	PCR Forward Primer (10µM)	0.4µl			
	PCR Reverse Primer (10µM)	0.4µl			
	cDNA templates	4µl			
	ddH2O	5.2µl			
**PCR reaction conditions**			95°C	90s	1 cycle
			95°C	05s	40 cycles
			60°C	15s	
			72°C	20s	

2. Results

2.1 Key metabolite extraction results and analysis

2.1.2 Effect of flavonoid metabolites on flower color

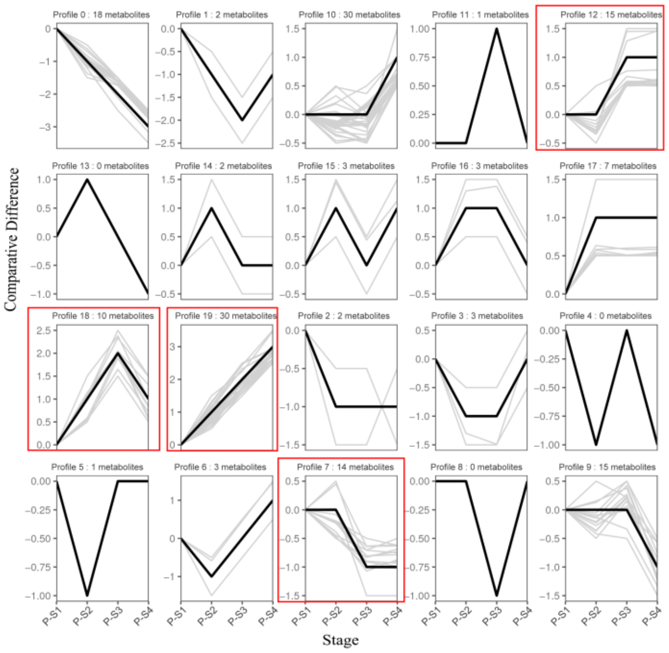

Figure S1. Trends of Flavonoid Differential Metabolites in Purple *Rhododendron latoucheae.*

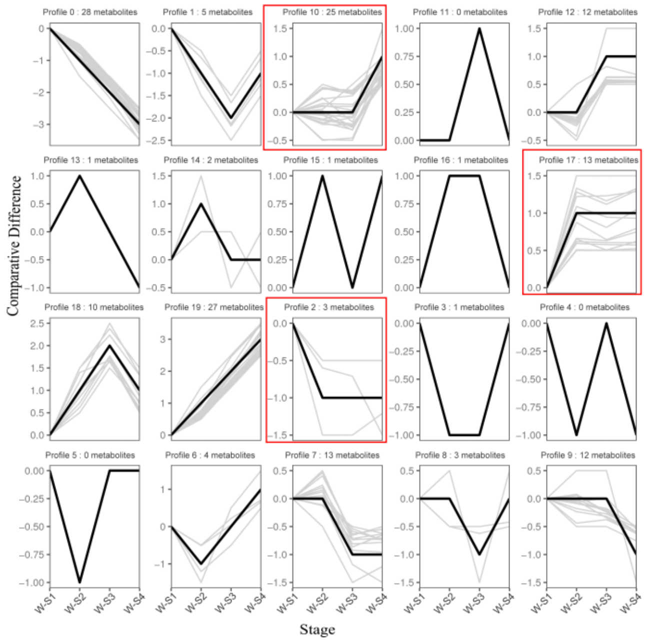

Figure S2. Trends of Flavonoid Differential Metabolites in White-pink *Rhododendron latoucheae.*

2.1.3 Effect of anthocyanins on flower color

Table S2. Vip and Log2(FC) statistics of Different Anthocyanins of *Rhododendron latoucheae.*

Period	Differential expression of anthocyanins	Vip	Log2（FC）	P-value
S1	Malvain-3-O-galactoside	1.332	1.559	0.039033
	Malvain-3-O-glucoside	1.250	1.853	0.028523
	Cyanidin-3,5-dio-O-glucoside	1.960	-0.708	0.028234
	Petunidin-3-O-glucoside-5-O-arabinoside	1.606	-0.888	0.027852
S2	Cyanidin-3,5-diglucoside	1.622	-0.461	0.013715
	Petunidin-3-O-glucoside-5-O-arabinoside	1.180	-0.58	0.016069
S3	Cyanidin-3-O-glucoside	1.714	1.025	0.001837
	Peonidin-3-O-glucoside	2.672	1.219	0.000120
	Malvain-3-O-galactoside	7.964	1.475	0.001915
	Malvain-3-O-glucoside	7.021	1.491	0.001788
	Peonidin-3-O-sambubioside	1.091	0.711	0.000569
	Petunidin-3-O-glucoside-5-O-arabinoside	1.392	-1.366	0.002296
	Peonidin-3,5-O-diglucoside	2.006	0.492	0.040067
S4	Peonidin-3-O-glucoside	1.701	0.769	0.009190
	Malvain-3-O-galactoside	6.932	1.638	0.001409
	Malvain-3-O-glucoside	5.897	1.686	0.000653
	Pelargonidin-3,5-diglucoside	1.541	-3.402	0.012412
	Malvain-3-O-rutinoside	1.807	1.715	0.004927

Note: S1 indicates the difference of anthocyanins between white-pink and purple in S1; S2 indicates the difference of anthocyanins between white-pink and purple in S2;S3 indicates the difference of anthocyanins between white-pink and purple in S3; S4 indicates the difference of anthocyanins between white pink and purple in S4.

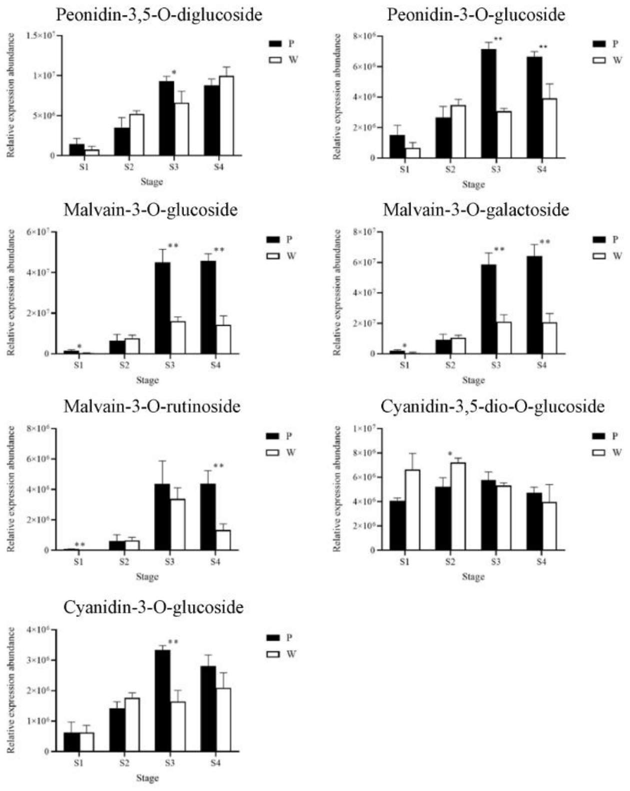

Figure S3. Anthocyanin content in *Rhododendron latoucheae.*

2.2 Key gene and key pathway assay results and analysis

Table S3. Composition of Bases and their Quality Values.

Sample Name	Raw data	High quality data	Efficient	Q20	Q30	GC
P-S1-1	60755986	60497876	99.58%	98.06%	94.43%	47.06%
P-S1-2	77351984	76977452	99.52%	97.99%	94.30%	46.87%
P-S1-3	87691336	87176030	99.41%	98.05%	94.43%	46.70%
P-S2-1	67875730	67585518	99.57%	97.87%	94.11%	47.45%
P-S2-2	79686488	79300920	99.52%	97.97%	94.29%	47.10%
P-S2-3	62984518	62592442	99.38%	97.99%	94.34%	47.09%
P-S3-1	63536168	63201344	99.47%	98.14%	94.69%	47.32%
P-S3-2	62874682	62492418	99.39%	97.99%	94.33%	47.26%
P-S3-3	71747226	71325652	99.41%	98.05%	94.48%	47.38%
P-S4-1	76461986	76099408	99.53%	97.95%	94.23%	47.92%
P-S4-2	71250166	70861956	99.46%	97.90%	94.06%	47.61%
P-S4-3	63961332	63594726	99.43%	98.07%	94.44%	47.62%
W-S1-1	74582028	74161362	99.44%	98.00%	94.31%	46.56%
W-S1-2	57008170	56666256	99.40%	98.02%	94.37%	46.81%
W-S1-3	68544564	68083132	99.33%	98.13%	94.63%	46.59%
W-S2-1	83038308	82591174	99.46%	97.94%	94.20%	47.30%
W-S2-2	92853624	92365018	99.47%	98.07%	94.50%	46.94%
W-S2-3	64931440	64583974	99.46%	98.07%	94.47%	46.52%
W-S3-1	76649534	76252436	99.48%	97.98%	94.32%	47.21%
W-S3-2	80612170	80076266	99.34%	97.96%	94.29%	47.20%
W-S3-3	66233998	65858110	99.43%	97.99%	94.34%	47.16%
W-S4-1	81079882	80731066	99.57%	98.06%	94.45%	47.23%
W-S4-2	63464100	63120286	99.46%	98.11%	94.56%	47.18%
W-S4-3	64368302	64097102	99.58%	98.14%	94.69%	47.45%

Note: The effective rate is the ratio of cleandata to the original data, and Q20 is the percentage of the number of bases with the quality value of sequencing bases above Q20 in cleandata; Q30 is the percentage of the number of bases whose sequencing base quality value reaches the level above q30 in cleandata; GC% is the percentage of GC content in cleandata.

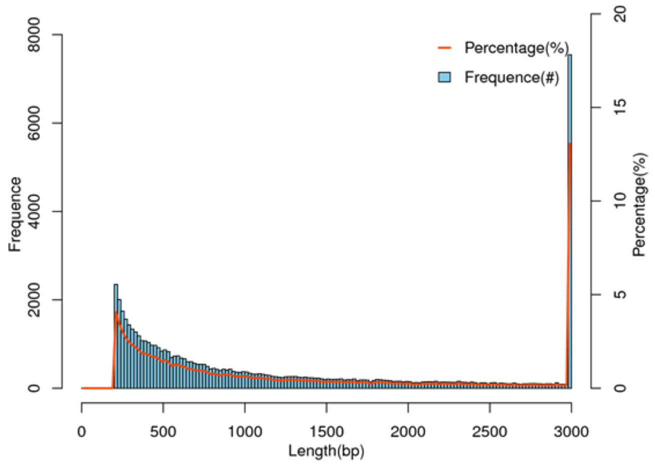

Figure S4. Unigene Length Distribution.

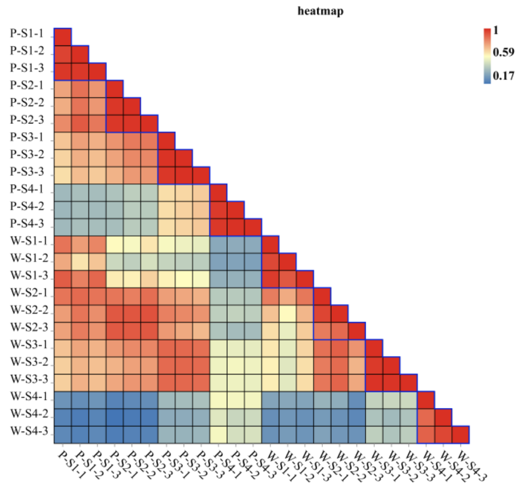

Figure S5. Heat Map of Correlation for Each Sample. The FPKM values were used to indicate the expression of each gene of *R. latoucheae*. And by calculating the pearson correlation coefficient of FPKM values of the samples, the correlation heat map of the samples was plotted.

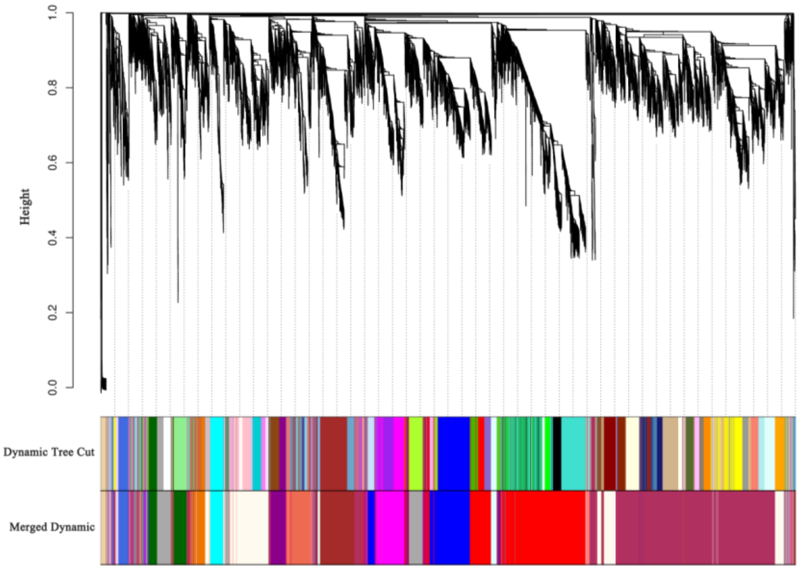

Figure S6. Module Level Clustering Diagram.

Table S4. Anthocyanin and Flavonoid Synthesis-related Unigene Log2 (FC) values.

ID	Symbol	S1(log2(FC))	S2(log2(FC))	S3(log2(FC))	S4(log2(FC))
Unigene0011575	CYP76C1	0.36	-0.88	-3.46	-3.93
Unigene0011577	CYP75B137	-1.57	-1.00	-1.34	0.46
Unigene0020093	TOGT1	-2.77	-1.87	-0.57	0.94
Unigene0011575	CYP76C1	0.14	-0.88	-3.46	-3.93
Unigene0018707	TKPR1	-0.99	-1.43	-0.78	-2.22
Unigene0023540	MYB12	0.38	8.99	4.83	3.15
Unigene0037903	CHS1	-1.67	-0.8	-1.38	-0.5
Unigene0039403	GT5	1.07	-2.33	-11.63	-13.18
Unigene0045101	F3H-2	-1.5	-0.77	-1.04	-0.08
Unigene0047699	CHS	-2.18	-1.77	-2.97	-1.05
Unigene0048513	RHM1	0.72	0.76	1.88	2.97
Unigene0036583	Os02g0190300	0.17	-1.28	3.2	1.84

Table S5. Heat Map of Correlation between Floral Traits and Each Module.

Module Name	L	a	b	c
MM.navajowhite2	0.030	0.105	-0.147	-0.082
MM.coral2	-0.306	0.485*	-0.506*	0.088
MM.magenta	0.150	0.177	-0.325	-0.265
MM.darkorange2	-0.253	0.082	0.054	0.195
MM.brown	-0.194	-0.308	0.397	0.286
MM.blue	-0.462*	-0.044	0.312	0.566**
MM.lightcyan1	-0.577**	0.195	0.014	0.127
MM.darkgreen	-0.55**	0.59**	-0.485*	0.264
MM.darkgrey	-0.179	0.32	-0.191	0.046
MM.cyan	0.469*	0.135	-0.287	-0.61**
MM.red	0.589**	-0.149	-0.148	-0.664***
MM.royalblue	-0.22	-0.234	0.35	0.218
MM.thistle1	-0.125	-0.206	0.35	0.31
MM.coral1	0.546**	-0.527**	0.472*	-0.06
MM.maroon	-0.095	-0.593**	0.817***	0.706***
MM.darkmagenta	0.546**	-0.726***	0.659	0.063
MM.floralwhite	0.179	-0.32*	0.430	0.245
MM.grey	-0.175	0.122	-0.019	0.039

Note: *indicates P < 0.05, * * indicates P < 0.01, *** indicates P < 0.001

Table S6. Unigene Related to Flavonoid in the Maroon Module.

Pathway	Gene ID	Gene Name
ko00941(Flavonoid biosynthesis)	Unigene0002596	*RlFAOMT*
	Unigene0012061	*RlCYP75A1*
	Unigene0016387	*RlANR*
	Unigene0027133	*RlLAR*
	Unigene0033582	*RlLDOX*
	Unigene0033619	*RlCHI3*
	Unigene0037903	*RlCHS1*
	Unigene0041684	*RlHST*
	Unigene0043745	*RlCHI*
	Unigene0045101	*RlF3H-2*
	Unigene0045102	*RlF3H*
	Unigene0046184	*RlF3H-2*
	Unigene0046185	*RlAN3*
	Unigene0046651	*RlFLS*
	Unigene0047699	*RlCHS*
	Unigene0047773	*RlCYP75A1*
	Unigene0052283	*RlFLS*
	Unigene0053894	*RlCHS1*

2.3 Metabolite regulation analysis

Table S7. The Correlation of MYB1, MYB3, MYB4, MYB12 and Key Metabolites.

Genes	Metabolites	Metabolite Class	Correlation coefficient	p-value
MYB1	Malvain-3-O-glucoside	Anthocyanidin	-0.70	0.000163
	Malvain-3-O-galactoside	Anthocyanidin	-0.69	0.000179

	Dihydromyricetin	Flavanonol	0.79	0.000005
	Peonidin-3-O-glucoside	Anthocyanidin	-0.71	0.000104
	Gallocatechin	Flavanoid	0.63	0.001104
	Malvain-3-O-glucoside	Anthocyanidin	-0.53	0.007198
MYB3	Malvain-3-O-galactoside	Anthocyanidin	-0.54	0.006355
	Dihydromyricetin	Flavanonol	0.72	0.000065
	Peonidin-3-O-glucoside	Anthocyanidin	-0.58	0.003103
	Malvain-3-O-glucoside	Anthocyanidin	0.64	0.000790
MYB12	Malvain-3-O-galactoside	Anthocyanidin	0.68	0.000266
	Peonidin-3-O-glucoside	Anthocyanidin	0.55	0.005056

Table S8. The Transcription Factors *RlMYB12*, *RlMYB1*, *RlMYB17*, *RlMYB315*, *RlbHLH93* and *RlbHLH96* are Correlated with Key Metabolites.

Genes	Metabolites	Correlation coefficient	P value
RlMYB17	Dihydromyricetin	0.75	0.000026
	Gallocatechin	0.67	0.000378
RLMYB315	Malvain-3-O-glucoside	-0.75	0.000028
	Peonidin-3-O-glucoside	-0.74	0.000035
RlMYB12	Malvain-3-O-glucoside	0.68	0.000266
	Peonidin-3-O-glucoside	0.55	0.005056
	Malvain-3-O-glucoside	-0.70	0.000163
RlMYB1	Dihydromyricetin	0.79	0.000005
	Peonidin-3-O-glucoside	-0.71	0.000104
	Malvain-3-O-glucoside	-0.62	0.001266
RlbHLH93	Dihydromyricetin	0.78	0.000008
	Peonidin-3-O-glucoside	-0.73	0.000054
	Gallocatechin	0.56	0.004381
RlbHLH96	Dihydromyricetin	0.69	0.000176
	Peonidin-3-O-glucoside	-0.66	0.000501

3. Discussion

3.1 Key genes and key metabolite regulatory relationships

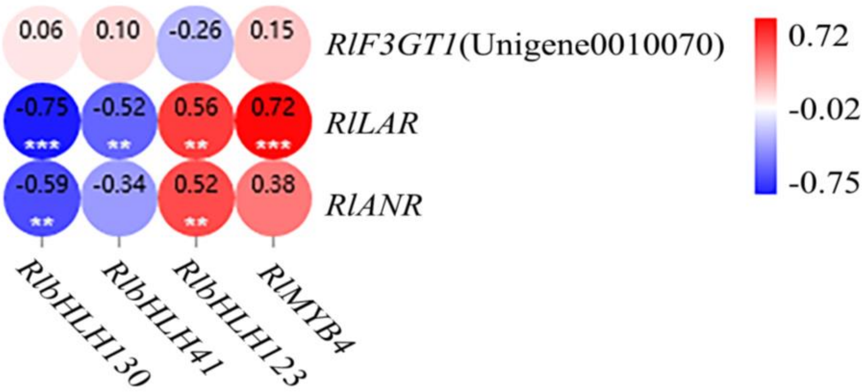

Figure S7. Heat map of Correlation between Key Structural Genes and Key Transcription Factors. note: *indicates P < 0.05, * * indicates P < 0.01, *** indicates P < 0.001.
